# Major Vault Protein (MVP) Associated With *BRAF* ^V600E^ Mutation Is an Immune Microenvironment-Related Biomarker Promoting the Progression of Papillary Thyroid Cancer *via* MAPK/ERK and PI3K/AKT Pathways

**DOI:** 10.3389/fcell.2021.688370

**Published:** 2022-03-31

**Authors:** Xubin Dong, Percy David Papa Akuetteh, Jingjing Song, Chao Ni, Cong Jin, Huihui Li, Wenjie Jiang, Yuhao Si, Xiaohua Zhang, Qiyu Zhang, Guanli Huang

**Affiliations:** ^1^ Department of Breast Surgery, The First Affiliated Hospital of Wenzhou Medical University, Wenzhou, China; ^2^ Department of Thyroid Surgery, The First Affiliated Hospital of Wenzhou Medical University, Wenzhou, China; ^3^ Department of Hepatobiliary Surgery, The First Affiliated Hospital of Wenzhou Medical University, Wenzhou, China; ^4^ Department of Pediatric Allergy and Immunology, The Second Affiliated Hospital and Yuying Children’s Hospital of Wenzhou Medical University, Wenzhou, China; ^5^ Children’s Heart Center, Institute of Cardiovascular Development and Translational Medicine, the Second Affiliated Hospital and Yuying Children’s Hospital of Wenzhou Medical University, Wenzhou, China; ^6^ Department of Thyroid Surgery, The Quzhou Affiliated Hospital of Wenzhou Medical University, Quzhou People’s Hospital, Quzhou, China

**Keywords:** major vault protein, papillary thyroid cancer, biomarker, tumor microenvironment, BRAFV600E, RAS

## Abstract

Papillary thyroid cancer (PTC) is the most common malignancy of the endocrine system, with an increase in incidence frequency. Major vault protein (*MVP*) is the main structural protein of the vault complex that has already been investigated in specific cancers. Yet the underlying biological functions and molecular mechanisms of *MVP* in PTC still remain considerably uncharacterized. Comprehensive analyses are predicated on several public datasets and local RNA-Seq cohort. Clinically, we found that *MVP* was upregulated in human PTC than in non-cancerous thyroid tissue and was correlated with vital clinicopathological parameters in PTC patients. *MVP* expression was associated with *BRAF* ^V600E^, *RAS*, *TERT*, and *RET* status, and it was correlated with worse progression-free survival in PTC patients. Functionally, enrichment analysis provided new clues for the close relationship between *MVP* with cancer-related signaling pathways and the immune microenvironment in PTC. In PTC with high *MVP* expression, we found CD8^+^ T cells, regulatory T cells, and follicular helper T cells have a higher infiltration level. Intriguingly, *MVP* expression was positively correlated with multiple distinct phases of the anti-cancer immunity cycle. *MVP* knockdown significantly suppressed cell viability and colony formation, and promoted apoptosis. In addition, downregulated *MVP* markedly inhibited the migration and invasion potential of PTC cells. The rescue experiments showed that *MVP* could reverse the level of cell survival and migration. Mechanistically, *MVP* exerts its oncogenic function in PTC cells through activating PI3K/AKT/mTOR and MAPK/ERK pathways. These results point out that *MVP* is a reliable biomarker related to the immune microenvironment and provide a basis for elucidating the oncogenic roles of *MVP* in PTC progression.

## Introduction

The thyroid is an important endocrine organ and a pathogenic target of human autoimmune diseases. Thyroid cancers are histologically subdivided into papillary (PTC), follicular, poorly differentiated, and anaplastic thyroid cancers (Landa et al., 2016). Plenty of studies had shown that the recurrence risk and cancer-related mortality of PTC patients were tightly associated with certain clinicopathological characteristics, such as the age of the first diagnosis, the size of the primary tumor, extrathyroidal invasion, and distant metastasis (Johnson et al., 1988; Asioli et al., 2010; Randolph et al., 2012). The overall prognosis of PTC patients was relatively satisfactory (Ghossein and Livolsi, 2008); however, patients with specific PTC subtypes (hobnail and tall cell variants) had poor prognoses (Yoo et al., 2016). Published studies had found that tumor-infiltrating immune cells (TIICs) could impact the response to immunotherapy, chemoresistance, and clinical outcome (Deschoolmeester et al., 2010). Therefore, analyzing the immunological function of the tumor microenvironment (TME), elucidating the molecular mechanisms of PTC development, and establishing promising immune-related biomarkers for PTC are crucial.

*MVP* is the main structural protein of the vault complex with a molecular weight of 110 kDa, which exists plentiful in the cytoplasm of eukaryotic cells (Vanzon et al., 2005). It was primarily reported to be involved in chemoresistance and to be significantly associated with the prognosis of various cancers, such as triple-negative breast cancer (TNBC) (Xiao et al., 2019) and liver (Losert et al., 2012; Yu et al., 2020), lung (Shen et al., 2019), and colon cancers (Henríquez-Hernández et al., 2012; Lötsch et al., 2013a; Teng et al., 2017). *MVP* also plays an essential role in multidrug resistance, autophagy, and signal transduction (van Zon et al., 2003). As MAPK-driven cancer, PTC mainly interacts with the mutually exclusive drivers (*BRAF* ^V600E^ and mutated *RAS*); PTC could be molecularly subdivided into *BRAF* ^V600E^-like (BVL) and RAS-like (RL) that represent differential regulation of the MAPK pathway and thyroid differentiation (Agrawal et al., 2014). Additionally, studies had also shown that *MVP* was correlated with signaling pathways related to drug resistance, such as PI3K/AKT and MAPK pathways (Park, 2012; Liu et al., 2019). However, there has been no related literature reporting the clinical and biological significance of *MVP* in PTC, and the potential relationship between *MVP* and TIME is yet to be determined.

In this study, we comprehensively made an inquiry from several aspects into an expression overview of *MVP* and its relationship with clinicopathological factors of PTC patients. Furthermore, we have conducted an in-depth exploration of TME through the analyses of the latest immune-related algorithms toward RNA-seq profiles. Significantly, we evaluated the potential clinical application value of *MVP* by analyzing the critical molecular characteristics of PTC. Our research results were broadly consistent in public databases and the local WMU-PTC cohort. In addition, based on functional enrichment analysis, we carried out a series of cellular and molecular assays to explore the connection between *MVP* and tumor immune-related molecular characteristics and to demonstrate the effects of knockdown and over-expressed *MVP* on PTC cell lines. The downstream regulatory mechanism of *MVP* was also preliminarily explored.

## Materials and Methods

### Datasets and Algorithms

Our study applied various public datasets of thyroid cancer. For the TCGA-THCA cohort, RNA-seq (RSEM normalization, level 3) and clinical profiles of thyroid cancer patients were acquired from the *TCGAbiolinks* package (Agrawal et al., 2014). This cohort included 501 PTC, 59 adjacent normal tissues (ANT), and 8 metastatic thyroid cancer samples (Supplementary Table S1, S2). The definition and data of progression-free survival (PFS) were acquired from TCGA-Clinical Data Resource (CDR) (Liu et al., 2018). Mutation status was acquired from VarScan2 MAF files of simple nucleotide variation from the GDC portal. Tumor mutation burden (TMB) was determined by the total number of non-silent mutations in specimens. MSI status (MANTIS score) for TCGA-THCA was used from a recent study (Bonneville et al., 20172017). The mRNA expression data and matched clinical information of GSE33630 (Dom et al., 2012), GSE60542 (Tarabichi et al., 2015), and GSE5364 (Yu et al., 2008) datasets were obtained from the GEO. In the present research, the Human Protein Atlas (HPA) immunohistochemistry images were used to explore the distribution and subcellular localization of *MVP*, comparing protein expression in malignant tissues and ANT (Uhlén et al., 2015; Uhlen et al., 2017). All IHC staining in the HPA project were performed using a standard protocol as described before (Kampf et al., 2012).

The “limma” R package was used to identify the differentially expressed genes (DEGs) between PTC subgroups. t-SNE was performed to explore the distribution of different groups using the “Rtsne” R package. The Spearman correlation analysis was evaluated by *cor. test* function in R. Genes and genes which had Spearman rho >0.5 or < −0.5, *p* < 0.001 with *MVP* were selected. Gene Ontology (GO) and Kyoto Encyclopedia of Genes and Genomes (KEGG) analyses were completed by *clusterprofiler* package. The Gene set enrichment analysis (GSEA) was carried out utilizing the hallmark and C6 oncogenic signature gene sets from the Molecular Signature Database (Subramanian et al., 2005). Significantly enriched gene sets were identified when the FDR-adjusted q value <0.25.

*ESTIMATE* package was used to quantify the immune score, stromal score, microenvironment score, and tumor purity (Yoshihara et al., 2013). For particular TIIC analyses, quanTIseq, CIBERSORT, and MCPcounter algorithms were performed to calculate the relative proportions of different immune components in TME. CIBERSORT is a deconvolution-based method for quantitatively estimating TIME contexture from RNA-seq data (Newman et al., 2019). MCPcounter is a computational approach based on RNA-Seq data of immune-specific marker genes (Becht et al., 2016a). quanTIseq is a deconvolution-based algorism to quantify the proportions of immune components from bulk RNA-sequencing data in TME (Finotello et al., 2019). Tumor ImmunoPhenotype (TIP) was an ssGSEA-based pipeline applied for cancer-immunity cycle profiling (Xu et al., 2018), which evaluates the relative activity of the main 7 steps of the cancer-immunity cycle.

### Patients, Thyroid Tissue Specimens, and RNA-Seq

79 pairs of thyroid specimens were derived from the Department of Thyroid Surgery, the First Affiliated Hospital of Wenzhou Medical University. Fresh tissues were snap-frozen in liquid nitrogen at the time of thyroidectomy and then stored at -80°C. Detailed clinicopathological information of WMU-PTC patients is summarized in Supplementary Table S3, S4. Details of the RNA-seq experimental protocol have been described in the previous publication (Dong et al., 2021). Briefly, total RNA was utilized to establish cDNA libraries for high-throughput RNA sequencing. The RNA expression proles were identified in the sequencing libraries obtained from an NEBNext Ultra RNA Library Prep Kit for Illumina (NEB, United States). The clustering of the sample was performed on a cBot Cluster Generation System using TruSeq PE Cluster Kit v3-cBot-HS (Illumina), and the library was sequenced on an Illumina NovaSeq platform. Due to the overall young age in our local cohort patients, to have more pT3/T4 and Stage IIIIV patients and better demonstrate the potential factors that affect the PTC progression, we evaluate our cohort with the seventh edition of the AJCC/TNM staging system (Lamartina et al., 2018).

### Cell Incubation and RNA Interference

HTori-3, BCPAP, TPC-1, and KTC-1 cells were all obtained from Shanghai Cell Biology, Institute of the Chinese Academy of Sciences (Shanghai, China). These cells were all cultivated in the RPMI 1640 medium (Gibco, United States) supplemented with 10% FBS (PAN Biotech, Germany). The genotypes of the PTC cell lines are shown in Supplementary Table S5. Lipofectamine RNAiMAX transfection reagent (Thermo Fisher Scientific, United States) was mixed with siRNA to transfect PTC cells. The sequences of the si-*MVP* were as follows: siMVP-1 sense: 5′- CCT​ACA​TGC​TGA​CCC​AGG​A -3′, siMVP-2 sense: 5′-ATC​ATT​CGC​ACT​GCT​GTC -3′, siMVP-3 sense: 5′- GCA​GAT​GAC​AGA​GGC​CAT​A-3′.

### Ectopic Expression

Full-length *MVP* cDNA was synthesized and inserted into pCDH-GFP +PURO-3xFlag and pCDH-GFP PURO vectors (Genepharma, Shanghai, China). The resulting vector or empty vector was transfected into PTC cells using Lipofectamine 2000 Transfection Reagent (Life Technologies, Carlsbad, CA) according to the manufacturer’s protocol. Infected cells were selected with puromycin (Invivogen) at 1 μg/ml.

### qRT-PCR and Primers

Total RNA was isolated from patient tissues and thyroid cancer cell lines by TRIzol reagent (Invitrogen, United States). All RNA specimens were temporarily stored at -80°C. The isolated RNA was analyzed at 260/280 nm, which ranges from 1.81 to 1.97. RNA reverse transcription was accomplished by the ReverTra Ace qPCR RT Kit (Toyobo, Japan). Real-time PCR was carried out and analyzed through an ABI 7500 System (Life Technologies, United States). The relative expression of *MVP* mRNA was presented using the normalized method of 2^−ΔΔCT^ with the endogenous control *GAPDH*. The primer sequences were as follows: *MVP* forward primer, 5′- CCC​AAC​ACT​GCC​CTC​CAT​CTA​AAG-3'; *MVP* reverse primer, 5′- ATC​TCC​ACG​ACC​TCC​ACT​TCC​TTC-3'; *GAPDH* forward primer, 5′- GTC​TCC​TCT​GAC​TTC​AAC​AGC​G-3'; *GAPDH* reverse primer, 5′- ACC​ACC​CTG​TTG​CTG​TAG​CCA​A-3'.

### Cellular Proliferation, Migration, and Invasion Assay

The CCK-8 (Dojindo, Japan) was employed to assess cell viability. Transfected PTC cells (1–1.5 × 10^3^/well) were seeded into 96-well plates. The optical density (OD) in each well was detected at 450 nm and recorded on a microplate reader (SpectraMax Plus 384, Molecular Devices Corporation, United States). For the colony formation assay, transfected PTC cells (1 × 10^3^/well) were seeded into a 6-well plate and then incubated for 5–8 days till clonogenicity. The plates were then softly washed by PBS and stained with crystal violet. Colony fields were calculated by the *ColonyArea* software (Guzmán et al., 2014). Transwell plates (Corning, United States) were applied to the migration test. Cells (4 × 10^4^ cells/chamber) were seeded onto the upper chamber, and the growth medium with 10% FBS was supplied to the bottom chamber. After culturing for 24 h in 37°C incubation, non-migrated cells in the upper chamber were cautiously wiped out using a cotton swab. Migrated cells were then fixed with methanol and stained with 0.1% crystal violet. For the invasion assay, the experimental procedure was similar to the migration test; in addition, the chamber is replaced by the Matrigel^®^ invasion chamber (Corning, United States). Migrated or invasive cells were photographed in a 10 × magnification microscope in at least 5 randomly selected fields for each well and counted using ImageJ software. For the wound healing assay, transfected cells were plated in 6-well plates, and the confluent monolayer was scratched after incubation for 24 h.

### Flow Cytometry Assay

The PTC cell lines were collected after being transfected for 72 h. Collected cells were washed with PBS in triplicate, and then resuspended with 300l 1binding buffer. Annexin V-fluorescein isothiocyanate (5l) and propidium iodide (5l) (BD Biosciences, United States) were added to 300l of cell suspensions at room temperature for 15 min in the dark. The apoptosis rate was defined as the percentage of Q2+ Q3 and analyzed by FlowJo (Tree Star, United States).

### Western Blotting

Transfected PTC cells were lysed in RIPA buffer (Solarbio, China), and phenylmethylsulfonyl chloride was used as a protease inhibitor to stabilize the whole lysate. The extracted proteins were quantified by the bicinchoninic acid assay (Thermo Scientific, United States). Then the proteins were separated by sodium dodecyl sulfate-polyacrylamide gel electrophoresis (BioRad, United States) followed by transferring them to the polyvinylidene difluoride (PVDF) membranes (Millipore, United States). The primary antibodies were as follows: *MVP* (16478-1-AP, Proteintech), phospho-AKT^Ser473^ (4060S, Cell Signaling Technology), total-AKT (4691S, Cell Signaling Technology), phospho-mTOR (381557, Zen Bioscience), total-mTOR (380411, Zen Bioscience), Phospho-p44/42 (4370T, Cell Signaling Technology), total-p44/42 (4695T, Cell Signaling Technology), phospho-p38 (4511T, Cell Signaling Technology), total-p38 (8690T, Cell Signaling Technology), and -Actin (AP0060, Bioworld Technology). Primary antibodies were used for immunoblotting at 1:1,000 dilution. The membranes were then incubated with a secondary antibody (ab97047 or ab6728, Abcam). Eventually, proteins were detected by the chemiluminescence kit (Thermo Scientific), and images of the protein bands were quantified by ImageJ software (NIH, United States).

### Statistical Analysis

Univariate and multivariate Cox analyses were used to search for independent risk factors. The MannWhitney test or Wilcoxon signed-rank test was adopted for comparisons between two groups. The KruskalWallis one-way analysis of variance (ANOVA) was used in multiple groups. The predictive performance of *MVP* was assessed by the receiver operating characteristic (ROC) curve analysis. The KaplanMeier curve analysis and log-rank test were applied to calculate the prognostic results. The best *MVP* critical point for KaplanMeier curves was determined by *res. cut* function. The CCK-8 assay was analyzed by two-way analysis of variance. In all experiments, at least three biological replicates were performed for each group. R 4.0.0 and Graphpad Prism 8.3.0 were recruited in our work.

## Results

### Major Vault Protein Was Upregulated in Papillary Thyroid Cancer

To identify molecules that are differentially expressed in BRAF-driver PTC, we analyzed TCGA-PTC datasets for gene expression differences between BVL and RL PTCs. The t-SNE analysis revealed that patients in two PTC subgroups were distributed in discrete directions (Supplementary Figure S1A). Notably, *MVP* mRNA was markedly higher in BVL-PTC than in RL-PTC (Supplementary Figure S1B,C).

**FIGURE 1:**
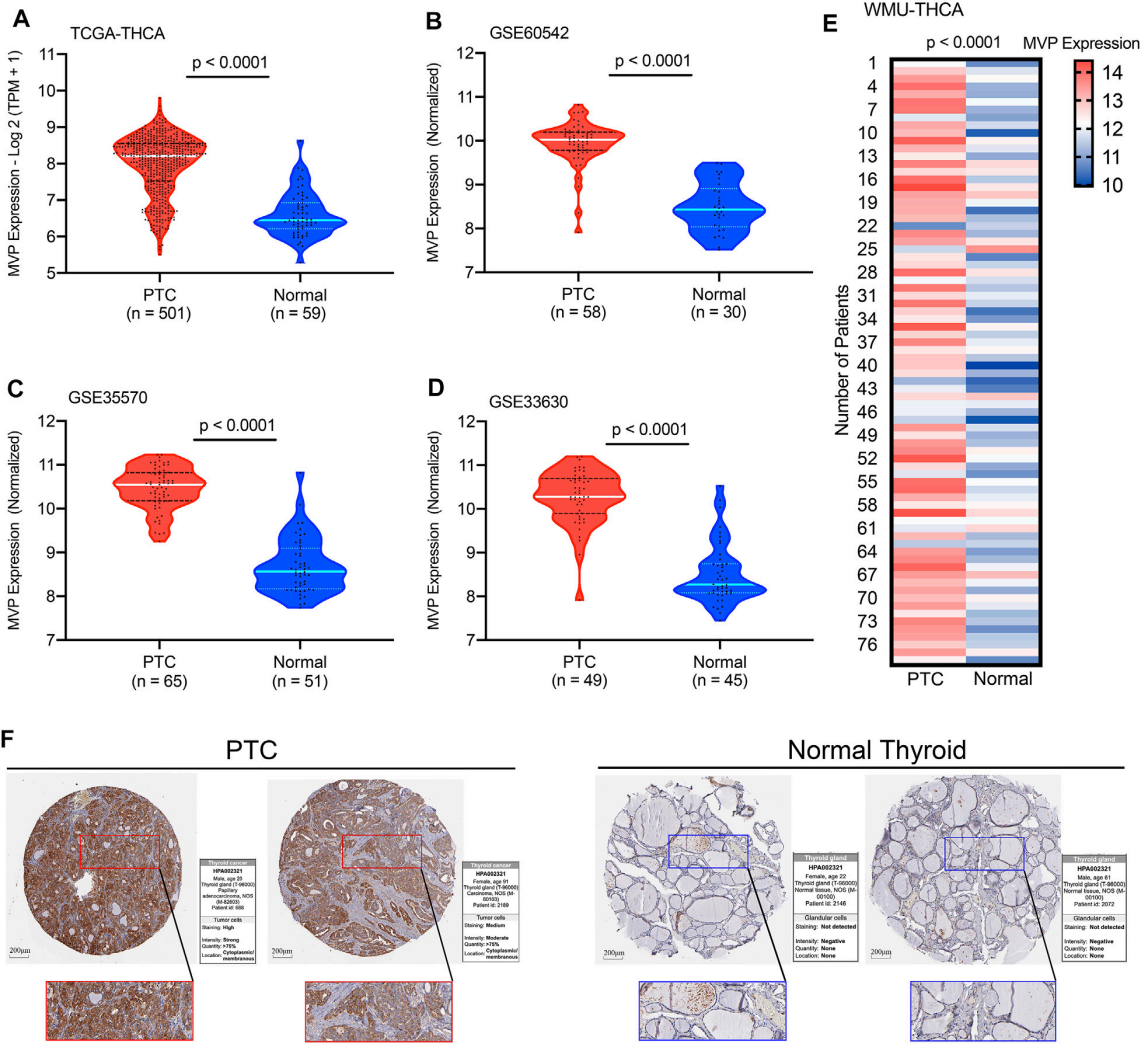
MVP expression levels in PTC and normal thyroid tissues. The *MVP* mRNA expression in PTC and ANT obtained from the **(A)** TCGA-THCA, **(B)** GSE60542, **(C)** GSE35570, and **(D)** GSE33630 datasets. **(E)** *MVP* expression in local WMU-PTC samples and matched ANT by RNA-Seq. **(F)** Representative IHC images and detailed MVP profiles in malignant tissues and ANT were acquired from THPA database.

For the patients in the TCGA-THCA database, the *MVP* expression in PTC tissues was significantly higher than that in ANT (Figure 1A, *p* < 0.0001). This result was consistent with the conclusions drawn from the GSE60542, GSE35570, and GSE33630 cohorts (Figures 1B–D, all *p* < 0.0001). To verify the *MVP* expression at the transcriptional level in the local PTC cohort (WMU-PTC), we sequenced 79 matched PTC and ANT, finding that the *MVP* expression in PTC was remarkably upregulated (Figure 1E, *p* < 0.0001). All the data mentioned above indicated that *MVP* was upregulated in PTCs. The representative immunohistochemical images and detailed information also verify that the *MVP* protein expression in PTC was upregulated (Figure 1F).

### Major Vault Protein Expression Was Positively Associated With the Progression of Papillary Thyroid Cancer

**FIGURE 2:**
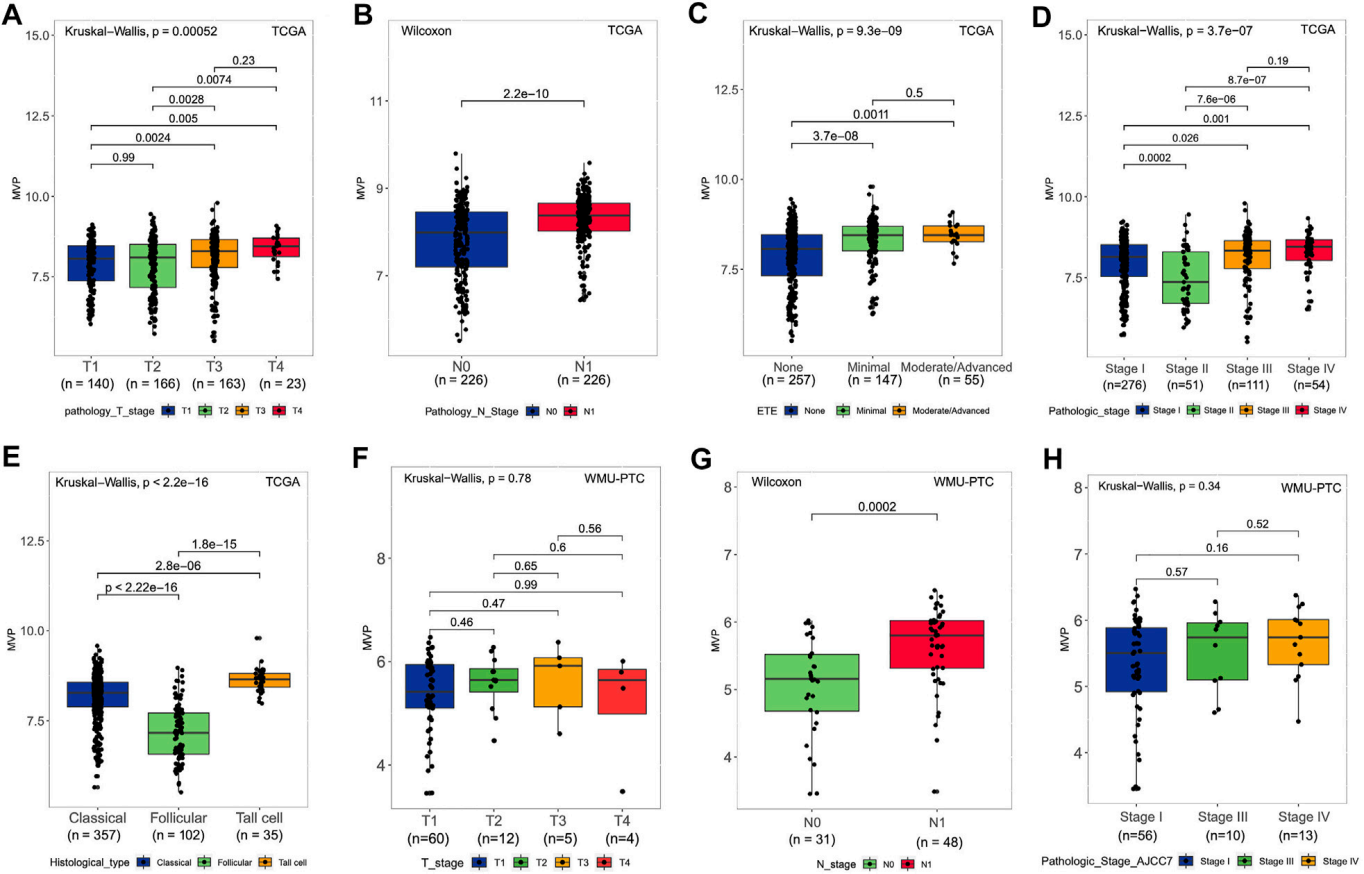
MVP level and relationship with clinical and pathological features of PTC patients. **(A-E)** For the TCGA cohort, the *MVP* level in distinct **(A)** T stage, **(B)** lymph node status, **(C)** extrathyroidal extension, **(D)** pathologic stage, and **(E)** histological subtypes. **(F-H)** For WMU-PTC dataset, *MVP* level in distinct **(F)** T stage, **(G)** lymph node status, and **(H)** pathologic stage.

The following analysis illustrated the relationship between *MVP* expression at mRNA level in PTC patients and clinicopathological characteristics in the TCGA dataset and our local patients. For the TCGA-THCA cohort, the *MVP* mRNA expression in PTC was considerably correlated with tumor size (Figure 2A, *p* = 0.00052), lymph node status (Figure 2B, *p* = 2.2e-10), extrathyroidal invasion (Figure 2C, *p* = 9.3e-09), and pathologic stages (Figure 2D, *p* = 3.7e-07). Moreover, the *MVP* level of follicular variant subtypes was lower than that of tall cell variant and classic subtypes (Figure 2E, *p* < 2.2e-16). We further analyzed the sequencing data of our local PTC patients, finding a positive relationship between the *MVP* level and LNM (Figure 2G, *p* = 0.0002). Still, there is no statistical difference between *MVP* expression and T stage (Figure 2F, *p* = 0.78) as well as disease stage (Figure 2H, *p* = 0.34).

### Association Between Major Vault Protein and Molecular Characteristics

**FIGURE 3:**
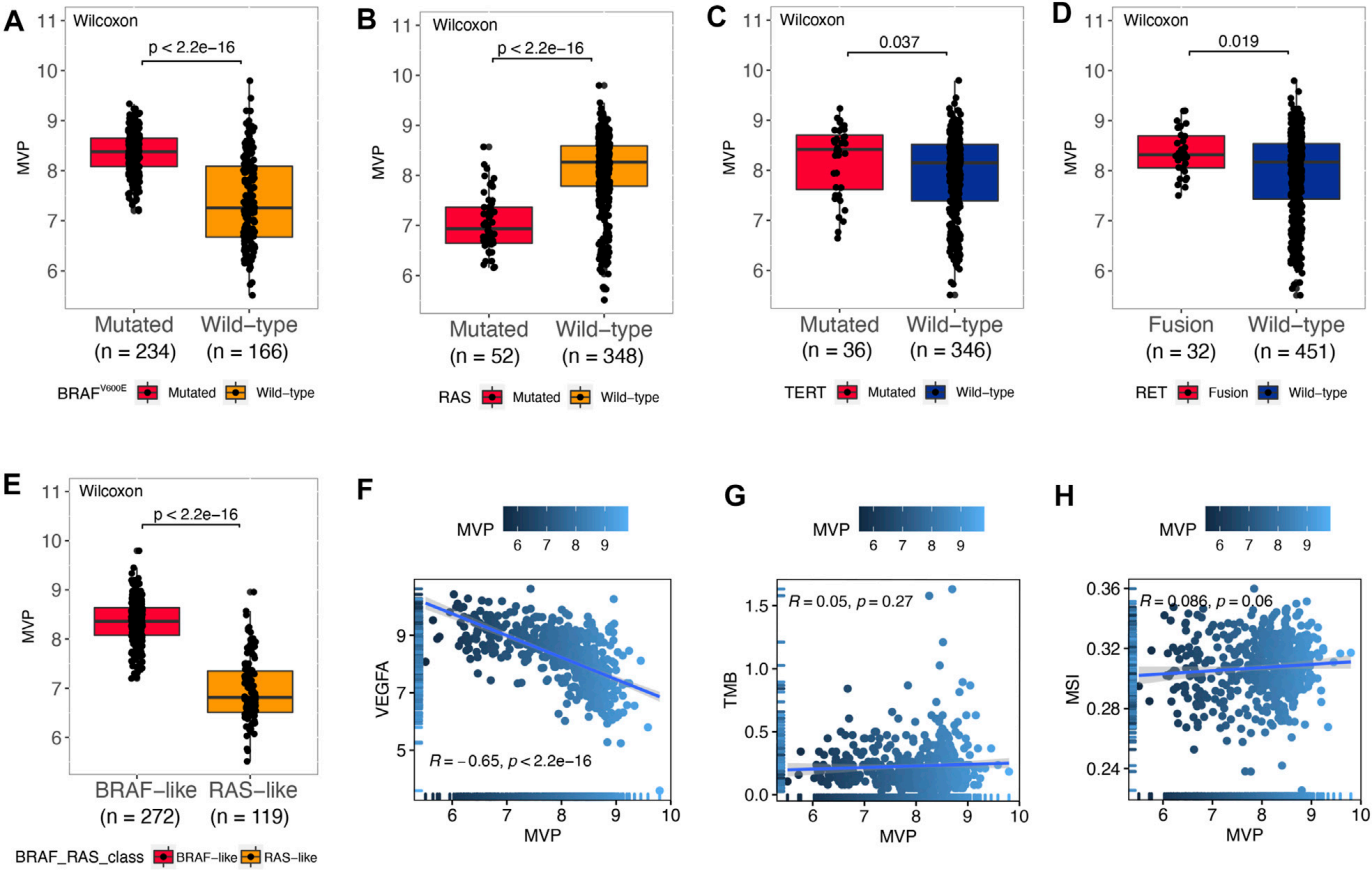
MVP was correlated with molecular characteristics in PTC. **(A–E)** *MVP* expression in PTC patients with different genotypic features: **(A)** *BRAF* ^V600E^ pattern, **(B)** *RAS* pattern, **(C)** *TERT* pattern, **(D)** *RET* pattern, and **(E)** BVL/RL. **(F–H)** Spearman’s correlations between *MVP* expression and **(F)** *VEGFA*, **(G)** TMB, and **(H)** MSI.

*BRAF* ^V600E^ caused persistent activation of the MAPK signaling pathway, resulting in unlimited cell proliferation and tumor formation (Xing, 2005). Moreover, a large number of studies had also authenticated that genes such as *RAS* (Liu et al., 2008), *TERT* (Xing et al., 2014), and *RET* (Romei et al., 2016) played a vital role in the tumorigenesis and progression of PTC. Therefore, we divided the patients based on driver mutation status and noticed that the *MVP* level was markedly increased in the *BRAF* ^V600E^ mutated group (Figure 3A, *p* < 2.2e-16) and *TERT* mutated group (Figure 3C, *p* = 0.037). Nevertheless, compared with the *RAS* mutated group, the *RAS* wild type has a higher level of *MVP* (Figure 3B, *p* < 2.2e-16). Additionally, compared with the *RET* wild-type subset, the *MVP* level in the *RET* fusion group was higher (Figure 3D, *p* = 0.019). According to 71 gene expression characteristics, PTC can be classified into BVL and RL (Cancer Genome Atlas Research, 2014). The *MVP* level in the BVL subgroup was considerably higher than that in the RL subgroup (Figure 3E, *p* < 2.2e-16). Nevertheless, there was no remarkable difference in the *MVP* mRNA level between *BRAF* ^V600E^ mutated and wild type in our local PTC patients (Supplementary Figure S2A). Interestingly, *MVP* had a strong negative correlation with *VEGFA* (Figure 3F, R = -0.65, *p* < 2.2e-16). On the contrary, *MVP* had no significant correlation with TMB (Figure 3G, R = 0.05, *p* = 0.27) and MSI scores (Figure 3H, R = 0.086, *p* = 0.06).

### Major Vault Protein Could Function as a Valuable Biomarker for Papillary Thyroid Cancer

**FIGURE 4:**
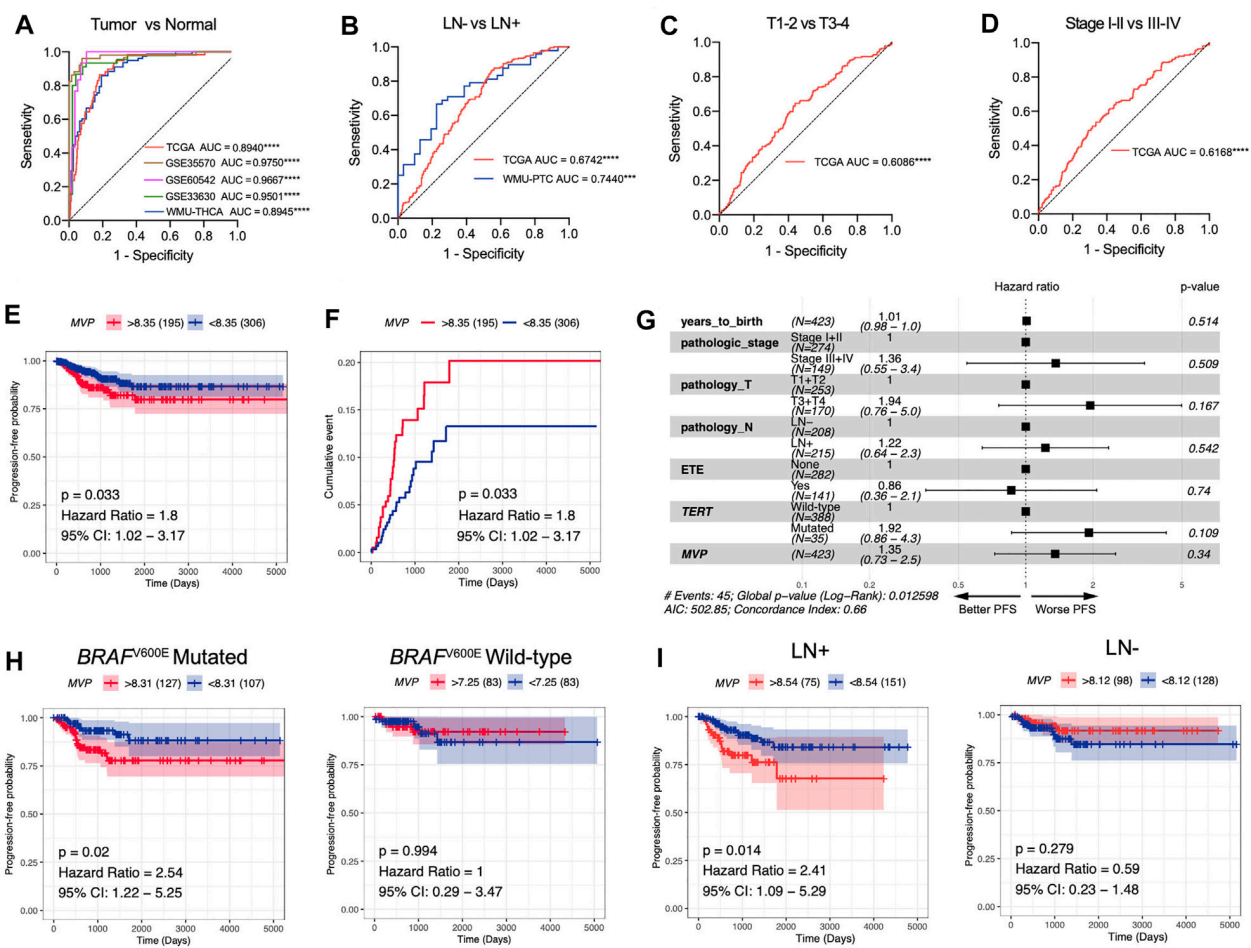
MVP is a promising diagnostic and prognostic factor in PTC. **(A)** ROC curve analysis revealed the diagnostic potential of *MVP* in PTC patients derived from public datasets and local WMU-PTC cohort. **(B-D)** ROC curve analysis exhibited the prediction capabilities of *MVP* in **(B)** LNM status, **(C)** tumor size, and **(D)** disease stage. **(E, F)** Survival curves contrast *MVP* ^high^ and *MVP* ^low^ patients together with the log-rank test. The p-value and hazard ratios (HR) with 95% confidence interval (CI) are calculated. **(G)** Multivariable Cox regression analyses of PFS in PTC patients. **(H, I)** Kaplan–Meier curves contrast *MVP* ^high^ and *MVP* ^low^ patients with different **(H)** *BRAF* ^V600E^ phenotype and **(I)** lymph node status. ^*^ *p* < 0.05, ^**^ *p* < 0.01, ^***^ *p* < 0.001, ^****^ *p* < 0.0001.

Taking the above findings into consideration, we conjectured that *MVP* might be a whole new biomarker of PTC. AUC values calculated through ROC curve analysis in TCGA, GSE35570, GSE60542, and GSE33630 databases, and our local validated cohort were 0.8940, 0.9750, 0.9667, 0.9501, and 0.8945, respectively (Figure 4A), which indicated that *MVP* had a diagnostic value. In addition, *MVP* also had predictive capability for LNM in the TCGA dataset (AUC = 0.6742) and local validation group (AUC = 0.7440, Figure 4B). For tumor size and disease stages, the AUC values of TCGA were 0.6086 and 0.6168, respectively (Figures 4C,D). Our results demonstrated that *MVP* might be a valuable disease biomarker of PTCs.

Then, we further speculated that *MVP* might be a promising prognostic predictor in PTC patients. Survival analysis revealed that high-level *MVP* was dramatically correlated with worse PFS in PTC patients (Figures 4E,F, HR: 1.8; 95% CI: 1.02–3.17; *p* = 0.033). By univariate Cox regression analysis, we determined that pathologic stages, T stage, extra invasion, and lymph node status were remarkably correlated with PFS (Supplementary Table S6). Yet the multivariate Cox regression analysis of PFS showed no independent prognostic factors (Figure 4G). Moreover, further survival analysis exhibited that high-level *MVP* was markedly correlated with shorter PFS in PTCs with *BRAF* ^V600E^ mutation or lymph node metastasis (Figure 4H, HR: 2.54; 95% CI: 1.22–5.25; *p* = 0.02; Figure 4I, HR: 2.41; 95% CI: 1.09–5.29; *p* = 0.014), but not in the *BRAF* ^V600E^ wild-type group or PTCs with negative lymph node metastasis. In our local WMU-PTC cohorts, survival analysis indicated that high *MVP* was associated with worse PFS (Supplementary Figure S2B, *p* = 0.13). The results indicated that *MVP* might affect the outcome of aggressive PTC patients.

### Predicted Functions and Pathways of Major Vault Protein

**FIGURE 5:**
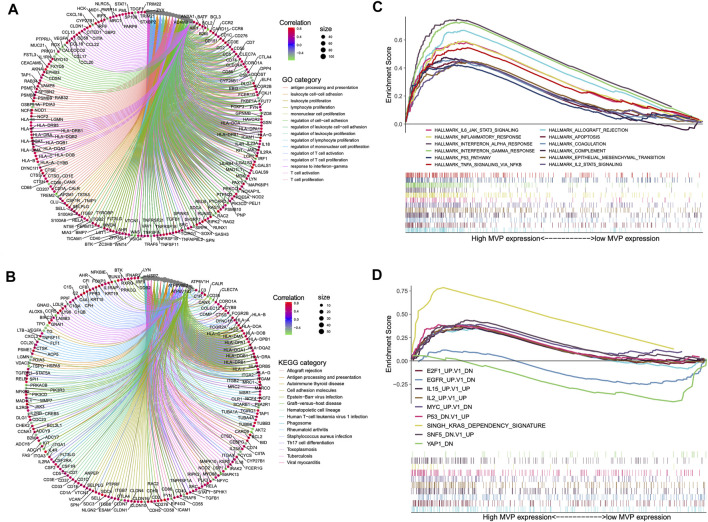
Summary of MVP co-expressed genes and functional enrichment analyses. Gene ontology **(A)** and KEGG pathways **(B)** were considerably related to *MVP* expression. Genes strongly co-expressed with *MVP* are exhibited in order of Spearman’s correlation coefficient. **(C)** GSEA presented crucial hallmarker terms enriched in high (up) *MVP* expression subgroup. **(D)** GSEA exhibited key oncogenic terms enriched in high (up) and low (down) *MVP* expression subgroup. The cutoff value between high (up) and low (down) level *MVP* is the median value in the cohort. Entire lists are shown in Supplementary Table S5–S7.

In particular biological circumstances, genes usually act as drivers to manipulate potential downstream pathways and trigger specific molecular functions (Jia et al., 2016). On this basis, we performed the co-expression analysis using the data of the TCGA cohort to clarify the genes that have intimately interacted with *MVP*. Subsequently, we picked out certain genes with significant co-expression correlation with *MVP* for GO and KEGG analyses (Figures 5A,B; Supplementary Table S7, S8). GO ontology and KEGG enrichment analyses showed that *MVP* was principally related to immune-related functions and the activation, adhesion, and proliferation of T cell. Additionally, GSEA analysis uncovered that the high-level *MVP* was mainly enriched in distinct oncogenic pathways and immune-related terms like IL-2 and IL-6 signaling, INF-α and INF-γ response, allograft rejection, and P53 pathway (Figures 5C,D; Supplementary Table S9). These results revealed that *MVP* might play an essential role in the progression and tumor-immunity process of PTC.

### Major Vault Protein Interrelated With Tumor-Infiltrating Immune Cells and Cancer-Immunity Cycle

**FIGURE 6:**
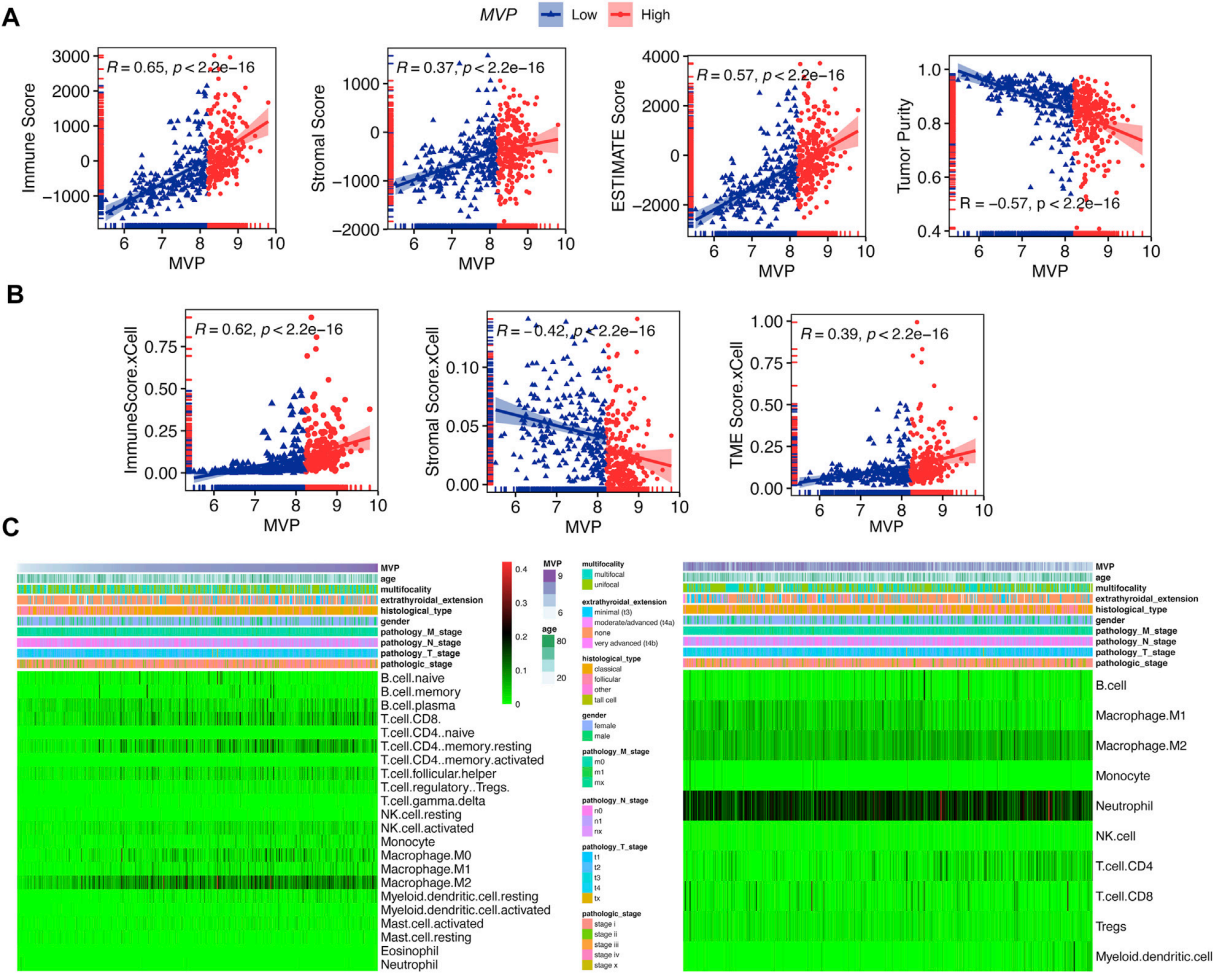
MVP interacted with TIME phenotype. **(A)** Spearman’s correlations between *MVP* level and immune, stromal, microenvironment scores, and tumor purity by ESTIMATE. **(B)** Spearman’s correlations between *MVP* level and immune, stromal, and microenvironment scores by xCell. **(C)** Profiles of TIICs in PTC immune microenvironment assessed through CIBERSORT and quanTIseq algorithms.

It has been expounded that TIICs widely existed in the TME of PTC and affected pathological processes, including tumorigenicity. Stromal and immune scores were used to describe the proportion of stromal and immune cells in the TME, respectively (Mao et al., 2013). PTCs in the TCGA database were subdivided into *MVP* ^high^ and *MVP* ^low^ sets according to the *MVP* median level. We quantitatively evaluated the immune and stromal infiltration by the ESTIMATE algorithm. We found *MVP* was correlated with the immune score (R = 0.65), TME score (R = 0.57), and tumor purity (R = -0.57) (Figure 6A). Similarly, utilizing another algorithm (xCell), we also found an apparent correlation between *MVP* expression and immune score (R = 0.65), as well as the TME score (R = 0.39) (Figure 6B). Interestingly, *MVP* expression was positively correlated with the ESTIMATE stromal score (R = 0.37) but negatively correlated with the xCell stromal score (R = -0.42). To sum up, the above analysis indicated that *MVP* had a positive relationship with the PTC immune microenvironment. With the purpose of further investigating the potential association between *MVP* expression and TIME, we established two different algorithms, CIBERSORT and quanTIseq, to calculate the abundance of TIICs. The clinicopathological features and infiltration level of TIICs based on the two algorithms are shown in Figure 6C.

**FIGURE 7:**
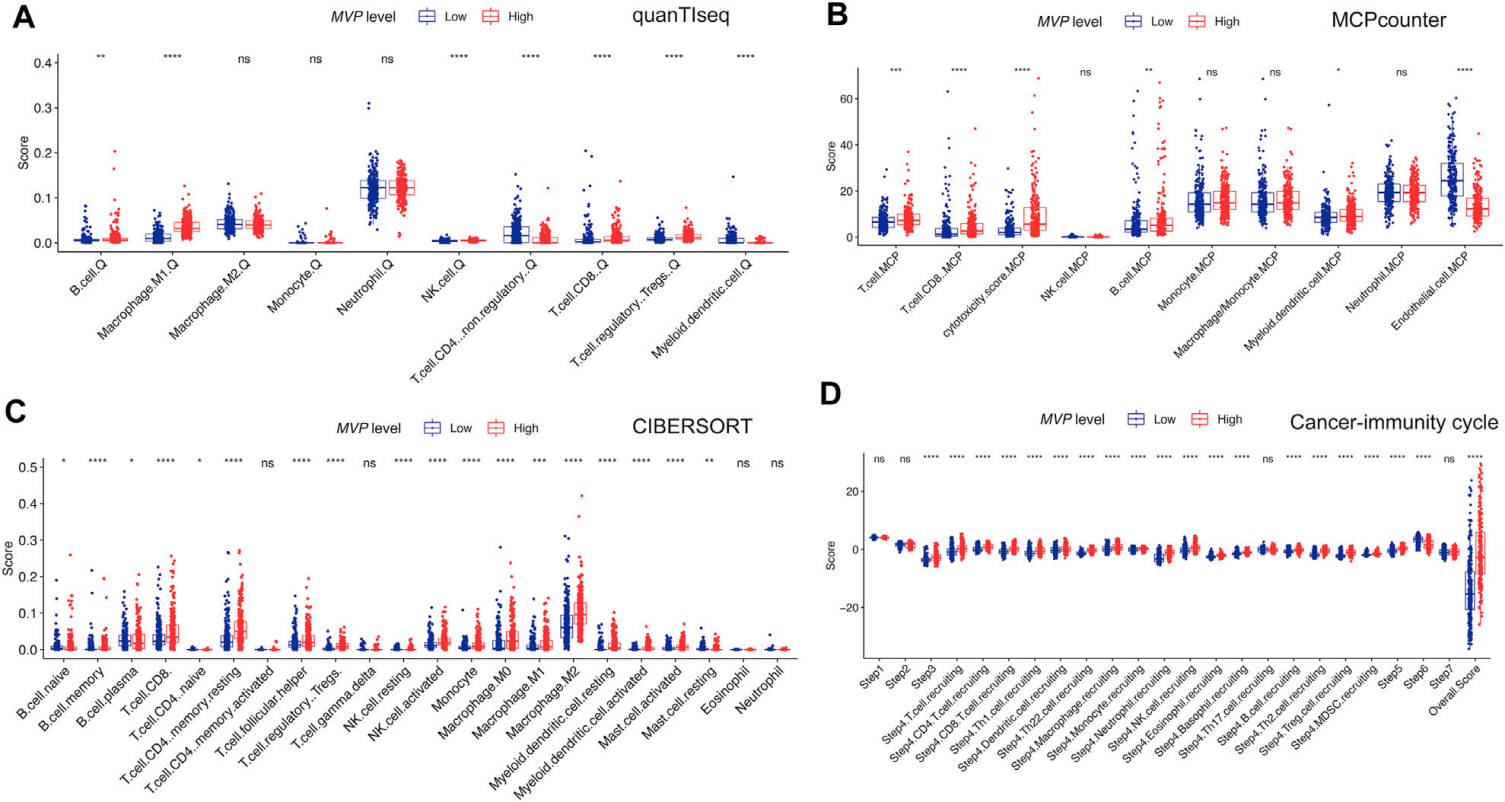
Profiles of immune cell infiltration in MVPhigh and MVPlow PTC from TCGA dataset. Compare the infiltrating immunocyte scores calculated by **(A)** quanTIseq, **(B)** MCP-counter, and **(C)** CIBERSORT in *MVP* ^high^ and *MVP* ^low^ groups. **(D)** Compare each step of activity of cancer-immunity cycle between *MVP* ^high^ and *MVP* ^low^ groups. ^*^ *p* < 0.05, ^**^ *p* < 0.01, ^***^ *p* < 0.001, ^****^ *p* < 0.0001.

Next, we attempted to determine whether the TIME changed in PTC samples with different expression levels of *MVP* (Figures 7A–C). By quantifying the immune infiltrating cellular component using three different algorithms (quanTIseq, MCPcounter, and CIBERSORT), we found that PTCs with high *MVP* expression were dramatically correlated with the high infiltrating level of anti-cancer CD8^+^ T cells and pro-cancer regulatory T cells. Simultaneously, more anti-cancer follicular helper T cells existed in *MVP* ^high^ PTC.

The cancer-immunity cycle could trigger successive molecular behaviors, which could initiate the anti-cancer immunologic reaction to executing cancer cells efficiently (Chen and Mellman, 2013). As exhibited in Figure 7D, killed tumor cells first release antigens, and the antigens are subsequently captured by antigen-presenting cells, which could present specific antigens to T cells and induce the priming and activation of effector T-cell responses (steps 1 to step 3). *MVP* ^high^ PTCs were more immunocompetent than *MVP* ^low^ in step 3. We noticed that *MVP* ^high^ PTCs had a significantly higher immune score in trafficking and infiltration of T cells to tumors (step 4 to step5). The *MVP* ^high^ group was significantly more active in the recruitment of CD4^+^ T cells, CD8^+^ T cells, Th1, dendritic cell, Th22, macrophage, monocyte, neutrophil, NK cells, eosinophil, basophil, B cells, Th2, and MDSC cells. Interestingly, we noticed that the process of recognition of tumor cells (step 6) was enhanced in *MVP* ^low^ PTC. Taken together, high *MVP* expression was connected with anti-cancer immune activity and was instrumental in the regulation of the cancer-immunity cycle.

### Deregulation of Major Vault Protein Promotes Papillary Thyroid Cancer Cell Proliferation, Migration, Invasion, and Apoptosis *In Vitro*

**FIGURE 8:**
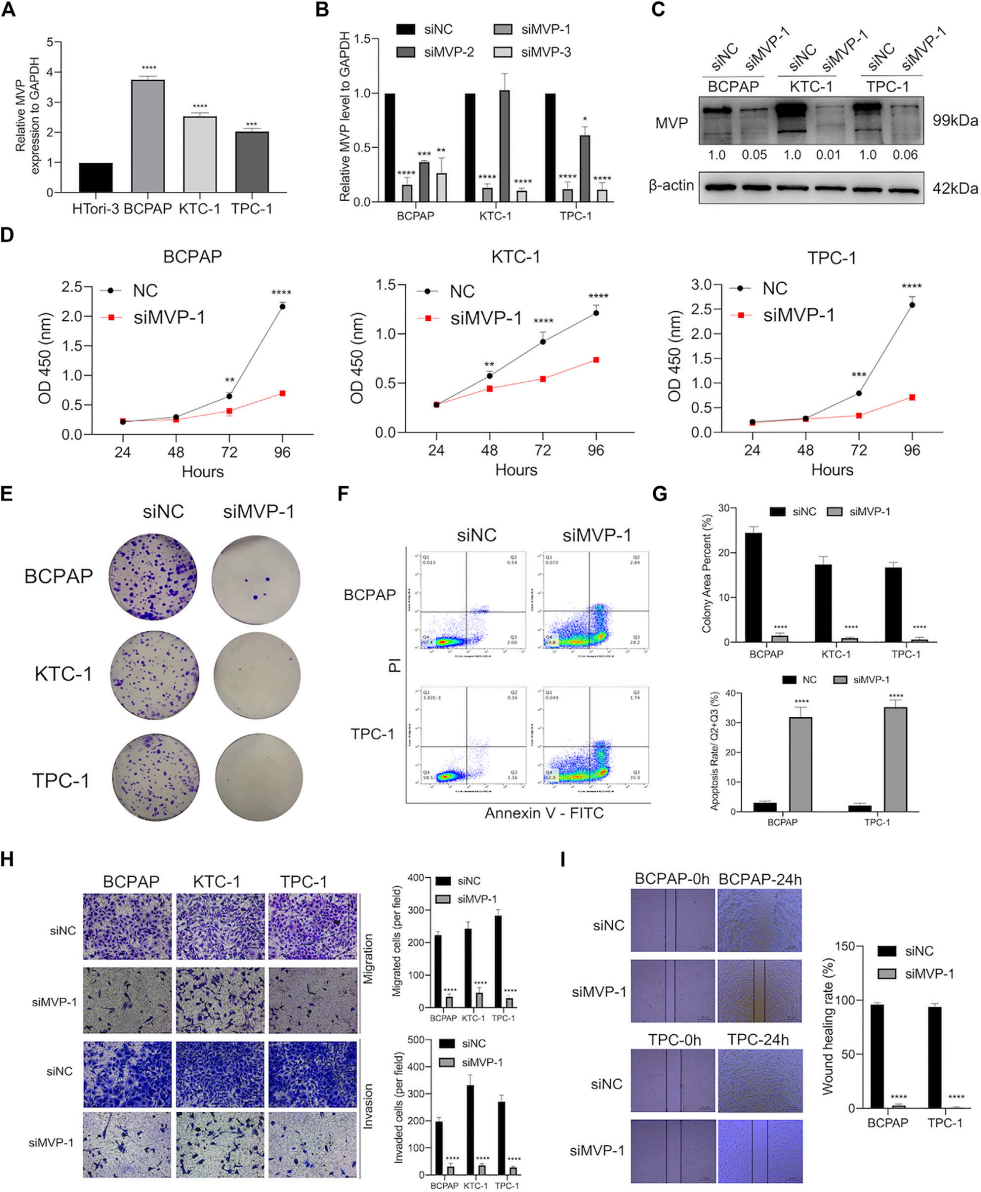
MVP was over-expressed in PTC cell lines, promoting the capability of proliferation, colony, migration, and invasion but attenuated apoptosis *in vitro*. **(A)** Relative transcript level of *MVP* was substantially higher in PTC cell lines (BCPAP, TPC-1, and KTC-1) than that in normal HTori-3 cell line. **(B)** Transcript levels of *MVP* in si-NC, siMVP-1, siMVP-2, and siMVP-3 groups were determined by qRT-PCR. **(C)** Protein levels of MVP in si-NC and siMVP-1 PTC cells were determined by Western blotting. **(D)** Cell proliferative capacity was detected through CCK-8 assays. **(E)** Clonogenic ability was determined through Colony formation assays. **(F)** Cell apoptosis was detected through flow cytometry. Early and late apoptotic cells were respectively characterized as PI^–^/Annexin V^+^ and PI^+^/Annexin V^+^. **(G)** Barplots showed repeated experimental results in Colony formation assays and flow cytometry. **(H)** Migration and invasion abilities were detected through Transwell assays. **(I)** Migration capability was determined by wound healing assays. All assays were independently repeated at least three times. Data are presented as the mean ± SD. ^*^ *p* < 0.05, ^**^ *p* < 0.01, ^***^ *p* < 0.001, ^****^ *p* < 0.0001.

As a validation of the above bioinformatic analyses, a series of cell biological experiments were designed and performed in three PTC cell lines *in vitro*. We observed that the *MVP* expression of PTC cell lines (BCPAP, KTC-1, and TPC-1) was significantly increased by contrast with normal HTori-3 cells (Figure 8A). After that, we attempted to elucidate the effects caused by deregulating *MVP*.

On the one hand, we already had demonstrated that *MVP* was correlated with tumor size and disease stage in PTC (Figures 2A,D,F,H). Aiming to figure out the role of *MVP* during the development of PTC, *MVP* knockdown experiments were performed, and three PTC cell lines were processed by siRNA targeting *MVP* (si-MVP). Through qRT-PCR evaluation at the mRNA level, we determined that the *MVP* transcriptional level was reduced in the PTC cell lines knocked down by si-MVP#1 (Figure 8B). Similarly, we determined that MVP protein activity in the PTC cell lines knocked down by si-MVP#1 was significantly decreased by Western blotting (Figure 8C). We observed that knockdown of *MVP* attenuated cell proliferation and defects in colony formation (Figures 8D,E,G). Using the Annexin V-PI assay to detect early and late apoptotic cells in the PTC cell population, we found an increased apoptosis rate in PTC cells after silencing *MVP* (Figures 8F,G).

On the other hand, we had demonstrated that the *MVP* level was correlated with LNM status and extrathyroidal extension in PTC (Figures 2B,C,G). Therefore, to explore the effect of deregulated *MVP* toward metastasis, a transwell assay was run to evaluate the migratory and invasive activity of PTC cells, which demonstrates that the migration and invasion abilities of PTC cells were hindered under *MVP* depletion (Figure 8H). Furthermore, it was proven by wound healing assays that silencing *MVP* restrained the migratory ability of PTC cells (Figure 8I). Our data revealed that *MVP* participated in regulating proliferation, migration, and invasion abilities of PTC cells *in vitro*.

### Major Vault Protein Promotes the Activity of MAPK/ERK and PI3K/AKT/mTOR Cascades

**FIGURE 9:**
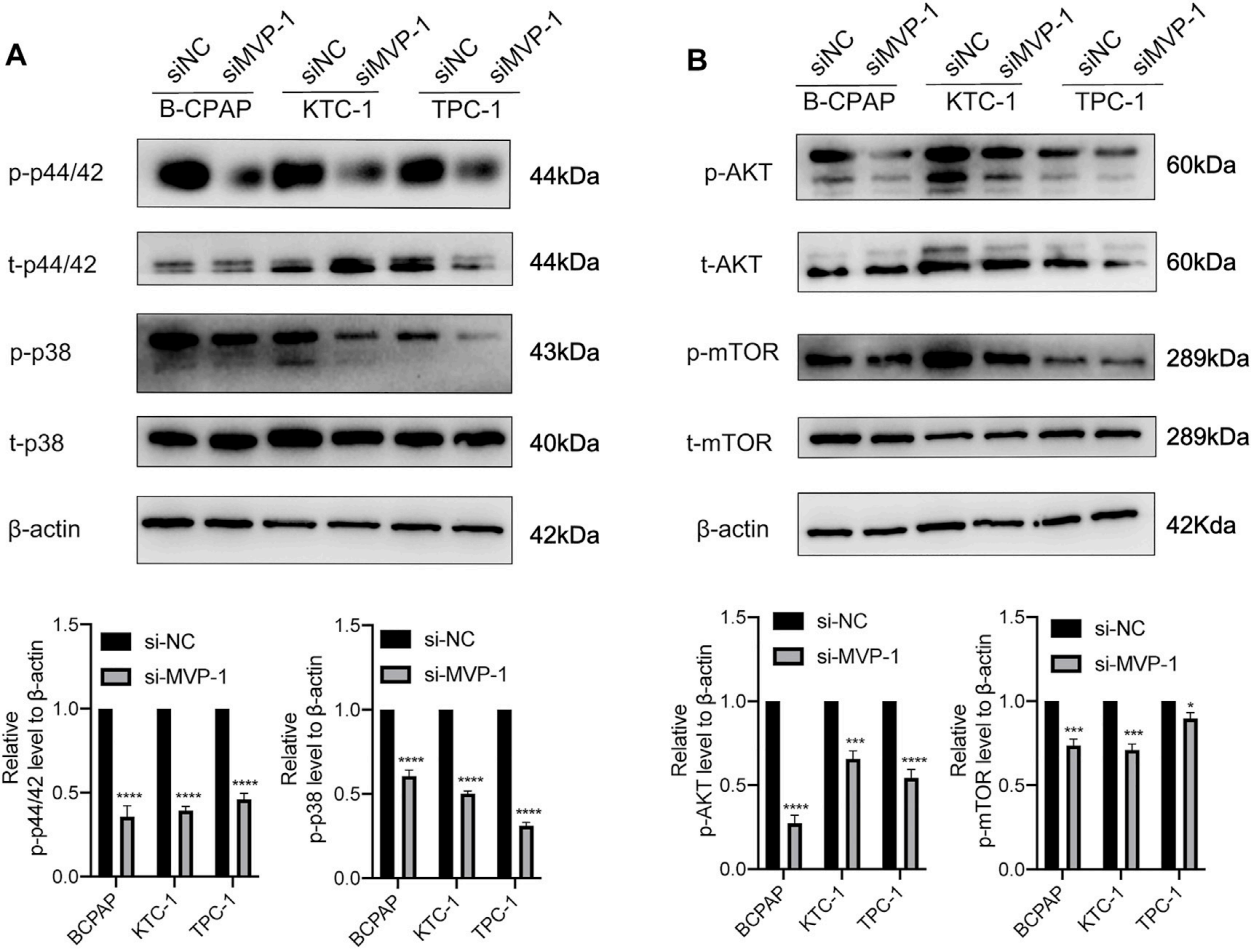
MVP promotes the activities of the MAPK/ERK and PI3K/AKT/mTOR cascades. The antibodies against MVP, phospho-p44/42 (p-p44/42), total p44/42 (t-p44/42), phospho-p38 (p-p38), total p38 (t-p38), phospho-AKT^Ser473^ (p-AKT^Ser473^), total AKT (t-AKT), phospho-mTOR (p-mTOR), and total mTOR (t-mTOR) were selected to evaluate the impact of *MVP* knockdown on the activities of **(A)** MAPK/ERK and **(B)** PI3K/AKT/mTOR cascades. GAPDH was used as the loading control. Representative images of Western blotting are showed in the upper panel. Quantitative analysis of Western blotting is displayed in the lower panel. All assays were independently repeated at least three times. Data are presented as the mean ± SD. ^*^ *p* < 0.05, ^**^ *p* < 0.01, ^***^ *p* < 0.001, ^****^ *p* < 0.0001.

Earlier research on mechanisms has shown that MAPK/ERK and PI3K/AKT/mTOR cascades functioned as regulatory effects in the development and pathogenesis of thyroid cancer. To illustrate *MVP* molecular functions on the above signal pathways, we further evaluated the activities of pathways through quantitative analysis of the images obtained by Western blotting. On the one hand, knocking down MVP in PTC cells inhibited activities of the MAPK/ERK pathway, characterized by reduced phosphorylation of ERK1/2 (p44/42) and p38 MAPK (Figure 9A). On the other hand, *MVP* knocking down in PTC cells reduced phosphorylation of AKT at Ser473 and mTOR (Figure 9B). These results suggested that *MVP* functions as an oncogene through promoting the activities of both MAPK/ERK and PI3K/AKT/mTOR signaling pathways.

### Rescue Experiment Indicated Major Vault Protein Maintains the Survival of Papillary Thyroid Cancer Cells

**FIGURE 10:**
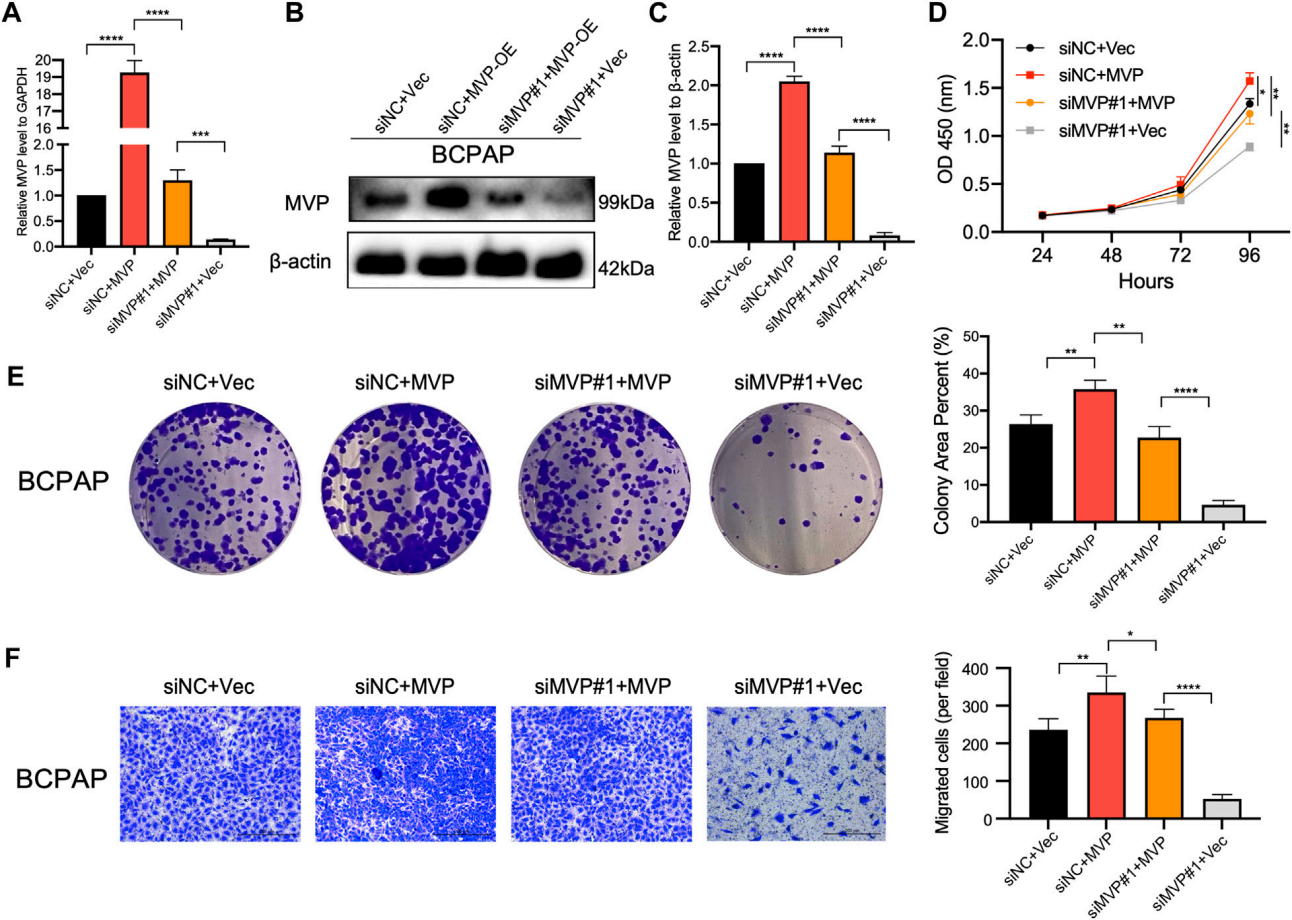
Rescue experiment indicated MVP maintains the survival and migration of PTC cells. **(A–C)** MVP expression was rescued in MVP silenced BCPAP cells. **(A)** qPCR and **(B, C)** Western blotting assays was performed to detect relative expression level of MVP. **(D, E)** CCK-8 and colony formation assays were performed to test cell proliferation of these BCPAP cells. **(F)** Transwell assays were performed to test cell migration activity of these BCPAP cells with restored expression of MVP. All assays were independently repeated at least three times. Data are presented as the mean ± SD. ^*^ *p* < 0.05, ^**^ *p* < 0.01, ^***^ *p* < 0.001, ^****^ *p* < 0.0001.

To further confirm the oncogenic role of *MVP*, the expression of *MVP* was rescued in BCPAP cells which had *MVP* silenced. First, qPCR and Western blotting assays showed both transcriptional (Figure 10A) and protein levels of *MVP* (Figures 10B,C) were rescued in *MVP* silenced BCPAP cells. Second, forced overexpression of *MVP* could significantly unmask the inhibition of cell survival (CCK-8 and colony formation assays) (Figures 10D,E). Forced overexpression of *MVP* in these initially silenced BCPAP cells significantly reversed the level of migration compared with the control group (Figure 10F). Taken together, the rescue experiments showed that *MVP* could maintain PTC cell survival and induce cells migration. Rescue experiments prove that si-MVP#1 is specific and there are no off-target effects.

## Discussion

The prognosis of most PTC patients is favorable with surgery and postoperative thyroxine replacement therapy. However, for dedifferentiated, recrudescent, and metastatic lesions, their median survival time is less than 6 months as radiotherapy and chemotherapy are the few treatment modalities available. A study had clarified a strong connection between TIME and immunotherapy in PTC (Na and Choi, 2018). Therefore, to ameliorate the prognosis of highly invasive thyroid cancer and develop more optimized treatment strategies, exploring the underlying signaling mechanisms becomes extremely urgent.

Vault is the largest cellular ribonucleoprotein complex with a hollow barrel structure, which comprises 3 protein components, namely, major vault protein (*MVP*), telomerase-associated protein 1 (TEP1), and vault poly ADP ribose polymerase (VPARP) (Park, 2012). Recent research works illustrate the vault is instrumental in a series of cellular processes, covering nuclear pore assembly, subcellular transport, signal transduction, and interferon response (Steiner et al., 2006; Berger et al., 2009; Ryu and Park, 2009; Vollmar et al., 2009). Recently, the literature has shown that *MVP* played an important part in cancer progression. *MVP* upregulation promotes oncogenesis and development of multiple tumor types (Xiao et al., 2019). Contradictively, a recent study showed that *MVP* inhibited lung cancer cell proliferation by suppressing the STAT3 pathway (Bai et al., 2019). In addition, *MVP* promotes glioblastoma survival and migration (Lötsch et al., 2013a), and inhibits apoptosis of human senescent diploid fibroblasts (Ryu et al., 2008) and human colorectal cancer cells (Ikeda et al., 2008). According to the information we have, this is the first work to dissect the clinical application value and potential molecular role of *MVP* in PTC from an entirely new perspective.

In this study, based on the analysis of the available data of 658 PTC patients derived from four public databases, we identified that *MVP* was significantly upregulated in PTC as compared to ANT. Subsequently, we took 79 paired PTC specimens and their corresponding ANT as a validation group and got the same conclusion by analyzing their RNA-Seq data. PTCs with a high pathologic grade or poorly differentiated still involve a certain amount of risk for disease recurrence and metastasis, and even endanger their lives (Farahati et al., 2004). Analysis of the TCGA database revealed that the increased *MVP* expression was consistent with more malignant clinicopathological features, involving larger tumor, more extensive LNM, and higher tumor stage, and extrathyroidal invasion implied that *MVP* might be bound up with the progression of PTC. In the local cohort, we also found that *MVP* is related to LNM.

According to the latest clinical diagnosis and treatment guidelines (ATA 2015) (Haugen et al., 2015), tissue/serum markers are not recommended for distinguishing benign and malignant thyroid nodules. The clinical differentiation of benign and malignant thyroid nodules mainly relies on pathological biopsy and tissue/cytological biopsy. A machine learning model has established a similar model on the TCGA database, and the discrimination between normal tissue and thyroid tissue exceeds 0.95 (Park et al., 2020). Our research group also published a model with good discrimination performance in identifying normal thyroid and thyroid cancer tissues (Wang et al., 2016). ROC curve analysis indicated that *MVP* had an accurate distinguishing ability for tumors in multi-center datasets (all AUC >0.89), but its actual ability to be used in the clinic still needs further research such as prospective clinical trials. In addition, *MVP* also had a specific predictive value for T-stage, LNM status, and disease stages of PTC patients in the TCGA cohort. In short, the above evidence revealed that *MVP* had the potential to serve as a promising biomarker of PTC.

*BRAF* ^V600E^ is a principal human oncogene originally reported in melanoma and incrementally discovered in other cancers, especially in PTC. Apart from clinicopathological characteristics, several genic features (such as *BRAF*, *TERT*, and *RET*) can be applied for risk stratification and prognostic scoring systems of thyroid cancer patients (Xing et al., 2005; Lee et al., 2007; Kim et al., 2012). PTC predominantly involves contradictory driving factors, and its comparative monotonicity of the genome brought about piles of studies which in detail analyzed the fundamental difference in genomics, epigenomics, and proteomics between BVL-PTCs and RL-PTCs. A study exposited that the existence of *BRAF* ^V600E^ mutation was the sole clinicopathological predictor of persistent disease after following up for 5 years (Elisei et al., 2012). *BRAF* ^V600E^ mutation is the most frequent mutation of *BRAF* and signifies high diagnostic accurate PTC, which can independently activate the downstream MER-ERK pathway and then lead to high MAPK signaling. A single mutation in *RAS* tends to indicate low risks, including benign nodules, NIFTP, and low-danger cancer. However, combined mutations in *RAS* usually mean increased malignancy. We found that *MVP* expression was elevated in *BRAF* ^V600E^ mutated, *RAS* wild-type, and BVL genotypes. We did not find the difference in the *MVP* level between *BRAF* ^V600E^ mutated and wild-type groups in our local PTC patients, which may be due to the low sample number in the wild-type group. *TERT* promoter mutations readily occurred in invasive thyroid cancer and were a strong predictor of poor clinical outcomes in thyroid tumor (Liu and Xing, 2016). *RET* fusions generally presented the BVL phenotype and upregulated the MAPK signaling (Mitsutake et al., 2006). We observed that the *MVP* level was substantially elevated in *TERT* mutated and *RET* fusion groups. Vascular endothelial growth factor (VEGF) promotes angiogenesis and endothelial cell proliferation, and is correlated with tumor growth and aggressive behavior of thyroid cancer (Salajegheh et al., 2011; Li et al., 2014). Correlation analysis of available RNA-Seq profiles suggested a strong negative correlation between *MVP* expression and *VEGFA*. Recently, Ronald J Hause et al. found MSI existed in varieties of cancers through whole-exome sequencing of 5,930 genomes from 18 types of cancers, particularly with regard to colorectal, endometrial, gastric, and thyroid cancers (Hause et al., 2016; Genutis et al., 2019). Earlier literature illustrated that cancers with high-frequency MSI often showed up as poor differentiation, expansive growth, histological heterogeneity, and high-level TIICs (Zheng et al., 2020). Interestingly, although SNPs in the *BRAF* and *RAS* were strongly associated with *MVP* expression, no significant correlation was found between *MVP* level and TMB and MSI scores in PTCs. Recently, research based on proteomic and bioinformatic analyses identified *MVP* as a prognostic biomarker for fatal prostate cancer (Ramberg et al., 2021). There were molecular characteristics that proved to be associated with *MVP* expression, and a high *MVP* level was associated with poor prognosis. However, we found *MVP* was not an independent prognostic factor of PTC. It might attribute to the favorable outcome of PTC patients, which causes the number of progression events to be small. These clinical analyses suggested that *MVP* may involve in the occurrence and progression of PTC.

Based on the transcriptional-level analysis, we then investigated the potential molecular function of *MVP* in PTC. Co-expressed genes commonly function coordinatively in biological molecular signals regulated by various factors and play a positive role in adaptive evolution (Niehrs and Pollet, 1999). GO ontology and KEGG enrichment analyses showed that *MVP* was correlated with the enriched immune-related genes. Hallmark terms related to immunologic processes or tumorigenesis pathways (e.g., IL-2 and IL-6 signaling, INF-α and INF-γ response, and P53 pathway) were enriched in GSEA analyses and associated with the high *MVP* level. A comprehensive dissection of TIME landscapes could contribute to identifying novel immunotherapeutic targets in PTC patients. Na et al. summarized immune cell abundances as an immune score to describe the profiles of the TIME in PTC (Na and Choi, 2018). Therefore, relying on two algorithms (ESTIMATE and xCell), we made use of transcriptional profiles of PTC tissues to quantify the immune and stromal proportions so that we can figure out the relationship between *MVP* and TME. Our research observed that the *MVP* expression level was positively related to immune scores, while stromal scores exhibited diametrically opposite results in two algorithms. In the light of the results of functional enrichment analysis, we found *MVP* might participate in regulating immune cell infiltration in PTC immune microenvironment.

Deconvolution algorithms are novel for decomposing heterogeneous cellular admixtures in the TME (Gentles et al., 2015; Orhan et al., 2020). In addition, MCP-counter quantifies the absolute abundance of 8 immune and 2 stromal cell clusters in heterogeneous tissues using transcriptome information (Becht et al., 2016b). Because of no criterion for assessing immune infiltration from RNA-seq profiles, we employed the deconvolution-based CIBERSORT and quanTIseq algorithms, and the marker gene-based MCP-counter algorithm to calculate the level of TIIC components quantitatively. Now there is evidence that high infiltration of CD8^+^ T cells was related to increased disease-free survival in thyroid cancer (Schreiber et al., 2011; Cunha et al., 2012). However, another retrospective study showed an opposite result that infiltration of CD8^+^ T cells was positively related to recurrence risk in differentiated thyroid cancer (Cunha et al., 2015). Recently, a study elucidated that immunotherapy was more effective against cancers with high CD8^+^ T-cell infiltration (Farhood et al., 2019). Our research found that *MVP* ^high^ status was associated with the increased number of anti-cancer CD8^+^ T cells, pro-cancer regulatory T cells, and anti-cancer follicular helper T cells in PTC.

The cancer-immunity cycle is a multi-step system in which the immune system distinguishes and eliminates tumor cells, and each step has corresponding positive or negative regulatory factors (Chen and Mellman, 2013). Immunotherapy is to activate the entire immune cycle by targeting these regulators at different phases and ultimately achieve the purpose of treatment. For example, the CTLA-4 inhibitor mainly acts on priming and activation (step 3), while PD-L1 and PD-1 inhibitors target step 7 (Chen and Mellman, 2017). Notably, our results showed that *MVP* ^high^ PTC exhibited higher overall anti-cancer immune scores in the cancer-immunity cycle. Furthermore, a variety of immune infiltrated cell components were increasingly identified in PTC with a high level of *MVP*. We also noticed that *MVP* ^high^ PTC was more vigorous in recruiting CD8^+^ T cells, CD4^+^ T cells, Th1, dendritic cells, Th22, macrophage, monocyte, neutrophil, NK cells, eosinophil, basophil, B cells, Th2, and MDSC cells, which was consistent with the alterations of TIICs in TIME. In summary, our findings elucidate that *MVP* may affect and regulate the cancer-immunity cycle.

Some studies reported that TIICs in TME might impact the proliferation, migration, and drug resistance of tumor cells (Gocheva et al., 2010; Joyce and Fearon, 2015). So far, there is no relevant research to determine the biological functions of *MVP* in PTC. Considering that transfection efficiency and genotypes varied among different cancer cells, we conducted a series of *in vitro* experiments after the knockdown of the *MVP* in three different PTC cell lines and expectedly elaborated that downregulated *MVP* inhibited the proliferation, migration, and invasion abilities of PTC cells. In the gain-of-function experiments, overexpression of *MVP* increases the proliferation and migration abilities of PTC cells. The effects of *MVP* knockdown on cell proliferation and migration were rescued in part by overexpression of *MVP*. In a nutshell, these lines of evidence demonstrated that *MVP* played a cancer-promoting role in PTCs.

Recently, the mechanisms of genetic events and cancer-related signaling pathways in thyroid cancer have attracted increasing attention. It has been clarified that MAPK/ERK and PI3K/AKT/mTOR cascades robustly participate in the tumorigenesis and development of thyroid cancer (Nikiforov and Nikiforova, 2011; Xing, 2013). Our findings showed that *BRAF* mutation was significantly correlated with *MVP* expression, whereas *RAS* mutation was connected with *MVP* ^low^ status. Several studies had demonstrated that MAPK/ERK and PI3K/AKT/mTOR pathways played critical roles in the tumorigenesis and progression of PTC. *BRAF* mutations primarily affected downstream signaling of the MAPK/ERK pathway, while *RAS* mutations mainly influenced the MAPK and PI3K/AKT/mTOR cascades (Nikiforov and Nikiforova, 2011; Xing, 2013). Therefore, to explore the cancer-promoting effect of *MVP* more deeply, we further discussed the impact of *MVP* in the above pathways. After the knockdown of *MVP*, we observed that the levels of p44/42 and p38 were obviously lower, indicating that *MVP* exerted its biological functions through the MAPK/ERK signaling pathway. A previous study on TNBC reached a similar conclusion as well (Xiao et al., 2019). Similarly, we observed that protein p-AKT^ser473^ and p-mTOR decreased after the knockdown of *MVP*, revealing that *MVP* may exert its effects by activating the PI3K/AKT/mTOR cascade. Our results were consistent with the relevant reports of *MVP* in other cancers (Lötsch et al., 2013b; Xiao et al., 2019). The further rescue experiments also validated that *MVP* could maintain PTC cells’ survival and induce PTC cells’ migration.

Several limitations in the current research still required to be considered. First, larger sample size information needs to be collected for verification of the association between *MVP* and clinicopathological tumor parameters, and also the prognostic value of *MVP*. After that, this study demonstrated that *MVP* interacted with anti-cancer immune response, neoplasm formation, and metastasis of PTC. However, potential *MVP*-related molecular functions in PTC progression require further exploration. *In vivo* experiments are necessary to further complement the regulatory mechanisms of *MVP*. Moreover, the gain-of-function experiments are also needed to explore if overexpressed *MVP* increases the proliferation and migration abilities in the HTori-3 cell line, which can help us understand the role of the *MVP* in PTC progression. In the end, the crosstalk between mutation drivers and altered tumor immune landscape in *MVP*-induced PTC lesions needs to be determined.

## Conclusion

Taken together, the biological effects and molecule mechanisms of *MVP* in PTC seem to be complicated. This work is the first comprehensive research to determine the expression level, relationship with clinical parameters, pathological characteristics, molecular hallmarks, TIICs, and cancer-immunity cycle functions of *MVP* in PTC. Our work first revealed that *MVP* is a reliable immune microenvironment-related biomarker, which also functions as a potential oncogene in PTC through activating the PI3K/AKT/mTOR and MAPK/ERK cascades. More in-depth studies of the *MVP* will contribute to our understanding of the mechanism of PTC progression.

## Data Availability

The data that support the findings of this study have been deposited into CNGB Sequence Archive (CNSA, https://db.cngb.org/cnsa/) of China National GeneBank DataBase (CNGBdb) with accession number CNP0002696. The original contributions generated for this study are included in the article/Supplementary Material, further inquiries can be directed to the corresponding authors.

## Supplementary Material

Supplementary Figure 1 Identification of *MVP* as an important *BRAF*-driven oncogene in PTC. **(A)** t-SNE analysis of the TCGA cohort. **(B)** Volcano plot of differentially expressed genes between BVL and RL PTCs. **(C)** Heatmap of top 20 differentially expressed genes between BVL and RL PTCs.

Supplementary Figure 2 Association of *MVP* level with BRAFV600E and prognosis in WMU-PTC patients. **(A)** *MVP* level in PTC patients stratified by *BRAF* ^V600E^ status. **(B)** Survival curves comparing patients with distinct *MVP* expression for progression-free survival.

## Abbreviations

MAPK, mitogen-activated protein kinase; TME, tumor microenvironment; TIME, tumor immune microenvironment; TIICs, tumor-infiltrating immune cells; ESTIMATE, estimation of stromal and immune cells in malignant tumors using expression data; CIBERSORT, cell-type identification by estimating relative subsets of known RNA transcripts; MCP-counter, microenvironment cell population-counter; ssGSEA, single-sample gene set enrichment analysis; MSI, microsatellite instability; SNP, single-nucleotide polymorphism; TIP, tumor immunophenotype; PBS, phosphate-buffered saline; PI, propidium iodide; PCR, polymerase chain reaction; NC, negative control; CCK-8, Cell Counting Kit-8; LNM, lymph node metastasis; AUC, area under the curve; RSEM, RNA-seq by expectation-maximization; VEGFA, vascular endothelial growth factor A; MDSC, myeloid-derived suppressor cells; NIFTP, non-invasive follicular thyroid neoplasm with papillary-like nuclear features.
