# Supplementary figures and images for: Major Vault Protein (MVP) Associated With BRAF V600E Mutation Is an Immune Microenvironment-Related Biomarker Promoting the Progression of Papillary Thyroid Cancer via MAPK/ERK and PI3K/AKT Pathways
Source: Front Cell Dev Biol. 2022 Mar 31;9:688370. doi: 10.3389/fcell.2021.688370 (PMC9009514; doi:10.3389/fcell.2021.688370)

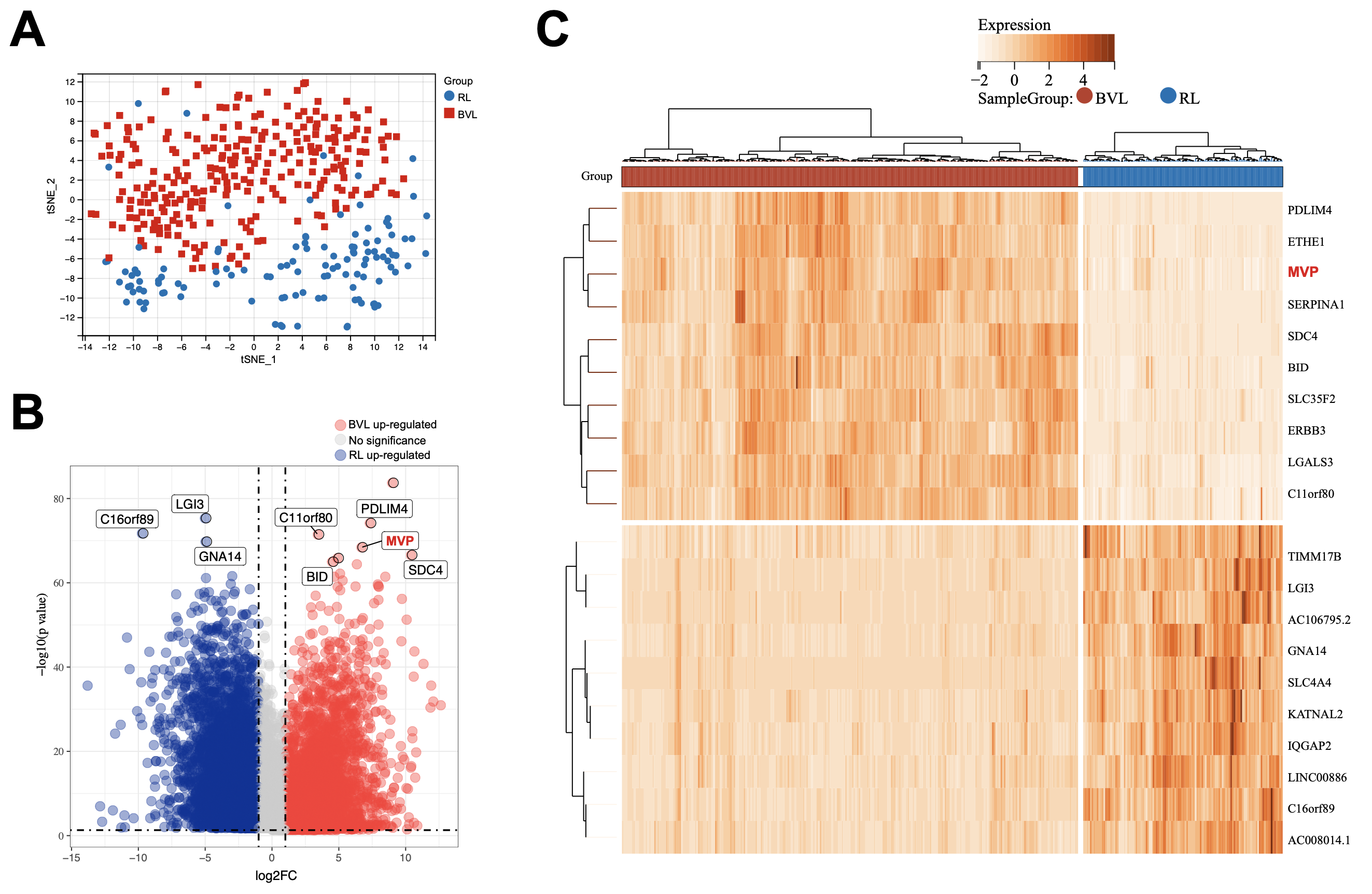

Supplement: Supplementary file 1 [file Image1.TIFF]
